# ‘A void in our community’: exploring the complexities of delivering and implementing primary care services for transgender individuals in Northern Ontario

**DOI:** 10.1017/S1463423624000203

**Published:** 2024-09-20

**Authors:** Erin Ziegler, Benjamin Carroll, Barbara Chyzzy, Don N. Rose, Sherry Espin

**Affiliations:** 1 Daphne Cockwell School of Nursing, Toronto Metropolitan University, Toronto, Canada; 2 School of Nursing, Queens University, Kingston, Canada

**Keywords:** mixed methods, normalization process theory, Ontario, primary health care, transgender persons

## Abstract

**Aim:**

To understand how the implementation of primary care services for transgender individuals is undertaken and delivered by practitioners in Northern Ontario.

**Background:**

Northern Ontario, Canada, has a shortage of primary care health practitioners, and of these, there are a limited number providing transgender primary care. Transgender people in Northern Ontario must also negotiate a lack of allied and specialty services related to transgender health and travel over long distances to access those services that do exist.

**Methods:**

A convergent mixed methods design was guided by normalization process theory (NPT) to explore transgender primary care delivery and implementation by nurses, nurse practitioners, physicians, social workers, and psychotherapists. A survey measuring implementation processes was elaborated through qualitative interviews with participants. Analysis of key themes emerging using the NPT framework informed understanding of primary care successes, barriers, and gaps in Northern Ontario.

**Findings:**

Key themes included the need for more education on transgender primary care practice, increased need for training and awareness on transgender resources, identification of unique gaps and barriers to access in Northern Ontario transgender care, and the benefits of embedding and normalizing transgender care in clinical practice to practitioners and transgender patients. These findings are key to understanding and improving access and eliminating healthcare barriers for transgender people in Northern Ontario.

## Introduction

*Transgender* describes an individual whose gender identity or expression differs from their assigned sex at birth (Institute of Medicine, 2011; Reisner et al., 2015). Language regarding gender identity has evolved, with terminology changing over time and between disciplines (Coleman et al., 2022; Thorne et al., 2019). The transgender community continues to experience discrimination, marginalization, and barriers to healthcare including lack of practitioners experienced and knowledgeable about healthcare needs, a deficiency of services, and structural barriers (Bauer et al., 2015a; Bauer et al., 2015b; Kinitz et al., 2021; Institute of Medicine, 2011; Müller, 2018).

Estimates of the transgender population vary. Canada is the first country to provide national census data about transgender people (Statistics Canada, 2022a). The recent Canadian census suggests 100 000 Canadians over the age of 15 identify as transgender, representing 0.33% of the population (Statistics Canada, 2022a); with estimates from 39 450 (Statistics Canada, 2022a) to 77 000 transgender adults living in Ontario (Giblon and Bauer, 2017). Estimates of Northern Ontario population of Two-Spirit, transgender and non-binary people are complicated by factors related to disclosure comfort and identity terminology (Statistics Canada, 2022a). However, a crude estimate based on 0.33% of the population of 790 000 people (Statistics Canada, 2022b) suggests 2600 transgender people live in Northern Ontario.

### Northern Ontario context

Northern Ontario is a region of over 860 000 square kilometers and consists of almost 80% of Ontario’s landmass with a population of approximately 80 000 people. Approximately 37% of those living in Northen Ontario live in rural areas (those having less than 1000 people), and another 25% live in small population centers (a community with more than 1000 but less than 30 000 people) (Statistics Canada, 2018). Northern Ontario comprises the north-eastern and north-western regions of Ontario, which extend north from Lake Huron and Lake Superior to Hudson Bay and James Bay, east to the Quebec boarder, and west to the boarder of Manitoba (Health Quality Ontario, 2017).

### Primary care

Primary care needs may include access to hormones therapy (Wylie et al., 2016), which has been identified as a priority need for the transgender population (Bourns, 2019; Heinz and MacFarlane, 2013). Access to a practitioner knowledgeable about transgender healthcare, who can provide hormone therapy is a known barrier to care (Bauer et al., 2015b; Cruz, 2014; Gardner and Safer, 2013; Heinz and MacFarlane, 2013; Roberts and Fantz, 2014). Educational programs for practitioners, including physicians and nurses, contain minimal transgender health-specific content (Chan et al., 2016; Dubin et al., 2018; Lim et al., 2015; Obedin-Maliver et al., 2011; Roberts and Fantz, 2014; Schreiber et al., 2021; Ziegler et al., 2022).

In Ontario, transgender healthcare is provided in primary care settings; however, there continues to be few practitioners providing care, long waiting times (Bauer et al., 2015b; Rotondi et al., 2013), and the need to travel based on geographic location (Dudar et al., 2018). Ziegler, et al. (2019; 2020a, 2020b) explored the delivery of care in Southern Ontario; however, understanding of care provision is lacking in Northern Ontario. Research conducted in rural Canada (Henriquez and Ahmad, 2021) and USA (Whitehead et al., 2016) highlighted increased levels of stigma, fewer supports and limited resources as key factors shaping access to primary care for LGBTQ populations.

The purpose of this study is to understand how primary care services for transgender individuals are implemented and delivered in Northern Ontario, identifying barriers and facilitators. Providing an understanding and increasing awareness of the implementation and delivery of primary care services may help to reduce the invisibility and disparities transgender individuals’ experience.

### Research questions

1. How is primary healthcare for transgender individuals implemented and delivered in Northern Ontario?
2. What activities do interdisciplinary team members engage in within the delivery of primary care to transgender individuals?
3. What has supported practitioners’ capacity to develop competence in delivering primary care to transgender individuals?

### Theoretical framework

The normalization process theory (NPT) is used to understand and explain the implementation of interventions in healthcare (May et al., 2011). NPT suggests that practices become routine or normalized as people work to enact them (May et al., 2009). Four theoretical constructs describe the work done to implement the change (May et al., 2011). *Coherence* explores how practices differs, individuals’ new roles, and values of the new practice. *Cognitive participation* promotes or inhibits the legitimation of the intervention; it is driven by participants’ commitments. The process of *collective action* is the material and mental work that is done to enact a practice. The final construct, *reflexive monitoring* is that patterns of collective action and outcomes that are continuously evaluated (May and Finch, 2009).

For this study, the intervention in healthcare explored using NPT was the delivery of primary care to transgender individuals. NPT was selected as a theoretical framework to guide this study as it will help to support the understanding of what was needed to implement this intervention in primary care, including the beliefs, behaviors, artefacts, and practices (May and Finch, 2009). NPT was used to guide the development of the semi-structured interview guide, survey and data analysis. Transgender primary care consists of any primary care services obtained by individuals who identify as transgender, including but not limited to general episodic care, chronic-care management, medical-supervised transition, and counselling.

## Material and methods

### Design

A convergent mixed methods design was used (Creswell and Poth, 2016; Creswell and Plano Clark, 2019). In this way, participant’s descriptions of the delivery and implementation of transgender primary healthcare could be explored by assessing ways in which qualitative and quantitative data converge (Creswell and Plano Clark, 2019). This study used data collection tools (modified Normalized MeAsure Development [NoMAD] survey (Finch et al., 2015) and semi-structured interview guide) previously published (REDACTED).

A purposive sampling strategy was used to identify and invite primary care practitioners in Northern Ontario providing primary care services to transgender individuals. Healthcare practitioners who were directly providing primary care services to transgender individuals were invited to participate in the study. Participants were asked to share the invitation to participate with other practitioners in their organization or who they knew who provided this care, utilizing a snowballing technique for recruitment. There remains a small network of practitioners providing this care, with many practitioners aware of each other allowing this to be a viable alternative method for recruitment. The purpose of examining participants already providing care to transgender individuals was to understand the characteristics of the patient population, the different roles, activities and preparation of practitioners to provide this care, the process of implementation, and factors which influence the program implementation.

### Data collection

Research ethics approval was received from a University Research Ethics Board. At the start of the interview, the interviewer reviewed the research purpose, consent, and interview process. Permission to audio-record the interview was obtained. Following this discussion, participants gave written consent using Google forms.

Open-ended, semi-structured interviews (*n* = 15) were conducted by Zoom teleconferencing between October 2020 and April 2021. Interviews ranged from 45 to 80 min were transcribed verbatim and reviewed for accuracy. The NoMAD survey (May et al., 2015), a tool to measure implementation processes, was modified to explore implementation of primary care services for transgender individuals. Surveys were distributed using Opinio (a web-based tool), the link to the survey was provided to each participant at the interview. A total of 13 surveys were completed (86.6% completion rate). A follow-up email was sent to all participants to remind them to complete the survey, and no further surveys were completed after the follow-up email.

### Data analysis

The survey data were analyzed using SPSS. For analysis and interpretation, descriptive statistics using means and standard deviation were used to describe participants’ responses (Normalization Process Theory, 2017). NVivo 12 was used for data analysis (QSR International, 2021), and all transcripts were double coded by two members of the research team. Deductive codes were initially developed from the concepts of NPT, research purpose, and research questions. Following this, inductive coding was done as new codes emerged during data collection (Miles et al., 2014). Line-by-line coding of all data sources was followed within the first-cycle coding (Miles et al., 2014). Second-level coding was completed by grouping first-cycle data into smaller categories, themes or constructs (Miles et al., 2014) within the large concepts from NPT. Codes that did not fit under NPT concepts were included. The code book was reviewed with the research team to obtain agreement on the organization of codes and naming of themes. Qualitative content analysis was used to summarize the informational content of the data (Sandelowski, 2000) and is a systematic and objective means of describing and quantifying phenomena (Elo and Kyngäs, 2008). Convergence of qualitative and quantitative data was completed (Creswell and Plano Clark, 2019) to give an overall understanding of how primary care services are implemented and delivered in Northern Ontario.

## Results

Fifteen individuals participated in the study. Varied primary care roles were represented, including a registered nurse (RN, *n* = 1), nurse practitioners (NP, *n* = 7), physicians (MD, *n* = 3), a pharmacist (Pharm, *n* = 1), and mental health clinicians which included either registered social workers or registered psychotherapists (MH, *n* = 3). Participants came from a variety of clinical settings across Northern Ontario and practiced in urban, rural and remote locations. Table 1 shows the self-identified comfort level providing primary care to transgender individuals.

**Table 1.** Practitioner comfort with transgender primary care practice table

Comfort level	*n* (missing)	*M (SD)*	Range
When you deliver primary health care to transgender patients, how familiar does it feel?^a^	13 (1)	8.7 (1.9)	3–10
Do you feel the delivery of primary health care to transgender patients is currently a normal part of the work you do?^b^	13 (1)	9.6 (2.3)	4–10
Do you feel the delivery of primary health care for transgender patients will become a normal part of your work?^b^	12 (2)	9.0 (0.6)	6–10

Note: *n* = 14.

a Level measured on an 11-point Likert scale where 0 = Still feels very new and 10 = Feels completely comfortable.

b Level measured on an 11-point Likert scale where 0 = Not at all and 10 = Completely.

The qualitative and quantitative results are presented with the four constructs of NPT. Data from the survey and interviews were converged and explored by construct. Survey questions related to NPT constructs were measured on a 5-point Likert scale where 1 = Strongly disagree and 5 = strongly agree. One item was reverse scored. Results of the quantitative data are presented in Table 2. Overall participants generally demonstrated positive attitudes toward providing care for transgender people, but reported they did not always understand the roles of other practitioners and staff, felt that was a lack of training about transgender healthcare needs and a lack of sufficient resources.

**Table 2.** NoMAD results

Item	Total *M (SD)* *N = 13(1)*	Prescribers NP/MD M(SD) *n* = 8	All other participants M(SD) *n* = 5
Coherence (Sense Making)			
1.1 - I can see how the delivery of primary care to transgender patients differs from usual primary care	3.77 (0.93)	3.87 (1.00)	3.60 (0.90)
1.2 - Staff in this organization have a shared understanding of the purpose of primary care for transgender patients	3.54 (1.20)	3.75 (0.89)	3.20 (1.64)
1.3 - I understand how the delivery of primary care to transgender patients affects the nature of my work	4.31 (0.48)	4.25 (0.46)	4.40 (0.55)
1.4 - I can see the potential value of primary care for transgender individuals for my work	4.77 (0.44)	4.75 (0.46)	4.80 (0.45)
Cognitive participation			
2.1 - There are key people who drive the delivery of primary care for transgender patients forward to get others involved	4.54 (0.66)	4.88 (0.35)	4.80 (0.45)
2.2 - I believe that participating in the delivery of primary care to transgender patients is a legitimate part of my role	4.85 (0.38)	4.63 (0.74)	4.80 (0.45)
2.3 - I’m open to working with colleagues in new ways to deliver primary care to transgender patients	4.77 (0.60)	4.88 (0.35)	5.00 (0.00)
2.4 - I will continue to support the delivery of primary care services for transgender patients	4.92 (0.28)	4.88 (0.35)	5.00 (0.00)
Collective action			
3.1 - I can easily integrate the delivery of transgender primary care into my existing work	4.69 (0.48)	4.50 (0.54)	5.00 (0.00)
3.2 - The delivery of transgender healthcare disrupts working relationships^a^	4.54 (0.66)	4.37 (0.74)	4.80 (0.45)
3.3 - I have confidence in other people’s ability to deliver transgender primary care	3.08 (0.76)	3.25 (0.71)	2.80 (0.84)
3.4 - Work is assigned to those with skills appropriate to the delivery of transgender primary care	3.62 (1.12)	4.00 (0.54)	3.00 (1.59)
3.5 - Sufficient training is provided to enable staff to delivery of transgender primary care	2.92 (0.86)	2.50 (0.76)	3.60 (0.55)
3.6 - Sufficient resources are available to support the delivery of transgender primary care	3.54 (0.97)	3.75 (1.04)	3.20 (0.84)
3.7 - Management adequately supports the delivery of transgender primary care	4.54 (0.52)	4.25 (0.46)	5.00 (0.00)
Reflexive monitoring			
4.1 - I am aware of reports about the effects of delivery of transgender primary care	3.69 (1.18)	4.00 (1.07)	3.20 (1.30)
4.2 - The staff agree that the delivery of transgender primary care is worthwhile	4.38 (0.65)	4.25 (0.70)	4.60 (0.55)
4.3 - I value the effects that delivering transgender primary care has had on my work	4.77 (0.44)	4.62 (0.52)	5.00 (0.00)
4.4 - Feedback about the delivery of transgender primary care can be used to improve it in the future	4.69 (0.48)	4.50 (0.54)	5.00 (0.00)
4.5 - I can modify how I delivery transgender primary care	4.38 (0.77)	4.00 (0.76)	5.00 (0.00)

a Item reverse scored.

### Coherence

Coherence, also known as ‘sense-making’, is the work that defines, organizes, and holds the practice together (May et al., 2009; Murray et al., 2010). For example, do participants have a shared sense of purpose? All participants discussed lack of access to primary care services in their community or geographic region for transgender individuals. One participant identified it as ‘*a void in our community*’ (NP4) and another commented that this ‘*is a highly underserved population*’ (MH3). RN1 described it as *‘something that we are lacking, especially where we live in Northwestern Ontario. There’s not a lot of services here for that. But it’s essential’*.

Participants agreed with the value of delivering care, viewing it as the same provided to all patients. ‘*I try to treat everybody pretty much the same…it’s not like I treat trans patients as they are rarefied…they are just people who need primary care* (MH3). Participants shared an understanding of the purpose of providing care to this population.

*It means providing services to trans people that not only meet their needs around social, legal or medical transition whenever that may be, but also meeting all the overall needs*(MH1).

Participants wanted to reduce existing barriers. *‘We don’t want people to not feel that they can get care because they can’t travel to [nearby city] or nobody’s really accepting patients’* (NP6) and ‘*providing care to our patients that might fall between the cracks’* (Pharm1). ‘*I think we recognize the vulnerability of the population*’ (MH3) and ‘*providing all-inclusive health care*’ (NP5) was a common theme among participants.

### Cognitive participation

Cognitive participation describes engaging and enrolling new people into the practice (May and Finch, 2009). Several participants discussed ways transgender primary care was initiated in their setting. Some were driven by community needs, including individuals *‘telling us what their needs are’* (MH3). One participant described their team support: *‘I got interested in trans health more because there seemed to be a need…everyone was super supportive’* (MD1).

Some participants realized that for their patients to access care they would have to learn, *‘we started to realize, we really don’t know what we are doing, so we had to, you know, get up to speed there’* (MH3).

*I talked with [my] patient and said ‘I have absolutely no experience but I will learn and if you are comfortable, we can try to learn together’ and they were like, ‘Yeah, let’s do it’ and that’s how I got started…I had a patient who had needs that needed to be filled* (NP2).

Participants were inspired to provide good care that countered anecdotal evidence of poor practice: *‘people sharing terrible experiences with doctors and then [thinking] “I can do better than that!”’* (MD1). In so doing, they reinforced that transgender care is primary care as described by this participant:

*I am a huge advocate of primary care…trans care is part of primary care and I think a lot of our job is to show primary care providers that there is nothing intrinsically challenging or complicated and [trans care] is so easily embedded in the primary care experience* (MD3).

Doing better included initiatives to support learning new terminology, *‘to adapt because the language is somewhat different’* (NP1) and develop inclusive practices, *‘working towards diversity so [administration] has even gone so far as putting together a self-learning package for all staff’* (NP3). These initiatives were described as efforts to realize the goal to *‘be able to eventually call it a positive space’* (MD2). Enrolling team members organizes a community of practice (May and Finch, 2009), *‘our entire team…are aware of the [trans patients] who have identified to us. So, they try to be careful to address them properly, and just to make them feel safer’* (NP6).

### Collective action

Collective action, the material and mental work, may be the reshaping of behaviors or actions or reorganization of a collective purpose (May and Finch, 2009). Despite needs in the communities and increased awareness and engagement of teams, it was still hard to integrate everyone into the provision of transgender primary care. ‘*So, we have five NPs and three physicians at the organization right now. And I’m the only one who is providing transgender care’* (NP4).

The majority of participants used guidelines and received mentorship to build competency and confidence. Most participants identified using Sherbourne’s *Guidelines for Gender-Affirming Primary Care with Trans and Non-Binary Patients* (Bourns, 2019), with three participants identified it as ‘*their bible*’. Access to a mentor or mentorship group was a key strategy to developing competency, ‘*there’s a primary care physician here that does trans care. So, he did say “call me anytime, if you have any questions”’* (NP6).

Despite engagement of practitioners, several barriers were identified including lack of funding, presence of transphobia and lack of professional development opportunities. Permanent funding was a struggle, ‘*our biggest barrier is funding*’ (MH1). Both obtaining funding, ‘*we had a hard time getting any funding really from the government when we first opened…that was a bit of a barrier for us’* (RN1) and renewing funding, ‘*most of our programs are not permanent full-time funding. So, we see programs and positions come and go rapidly*’ (MH2).

The presence of transphobia in the community was a challenge. ‘*We ran into some very blatant transphobia where they refused to house our program in their clinical site*’ (MD3) and ‘*that’s a really, really Christian community. I don’t think this person is going to be totally safe coming out as trans in that community because of, like the violence that happens there*’ (MD2).

Participants identified accessing professional development opportunities was a barrier due to geography. Travel is challenging given the distance and higher costs from this region. Limited funding further restricted attending professional development:

*They are no longer funding any out of province travel. So, before I was able to attend conferences that were key professional development…But now I can’t unless they’re in Ontario* (MH1).

### Reflexive monitoring

Reflexive monitoring allows for actions and outcomes to be continuously evaluated and reflected on (May and Finch, 2009; Murray et al., 2010). Informal or general feedback forms were used to evaluate services provided; however, no participants collected formal feedback specific to transgender patients. Participants discussed asking patients for feedback, stating ‘*we are always looking for feedback*’ (RN1) and ‘*we do have feedback surveys that are issued to patients and we just get feedback from everybody*’ (NP6). Participants talked about using waitlists as outcome markers, trying to have minimal waitlists for transgender patients, with appointments being booked within 1–6 weeks. Practitioners also identified transgender patients as a priority, stating ‘*trans people get front of the line here…they are a priority population*’ (MH3) and ‘*trans clients fall underneath our priority population*’ (NP5).

Participants spoke about self-appraisal, reflection and validation of their work. ‘*I definitely get a lot of fulfillments…providing care to my community*’ (MD2), and ‘*seeing the benefits in my patients I think really validates you as a provider*’ (NP6). Participants spoke about being proud of their work and doing it well, ‘*I think we’re doing pretty good*’ (NP5) and ‘*I get more joy out of the work I do with my trans patients than any other avenue or any other area that I work in*’ (NP4).

Most participants mentioned the need for transgender care to be integrated into ‘regular primary care’ to improve access and eliminate barriers. One participant stated ‘*everybody should be able to provide safe competent services to somebody who identifies as trans. It shouldn’t make a difference*’ (NP2). Participants also identified that providing care to this population was primary care and within the scope of primary care practitioners; ‘*I personally believe that trans related healthcare is something that is easily embedded into primary care*’ (MD3).

## Discussion

The purpose of this study was to understand the delivery of primary care services for transgender individuals in Northern Ontario. Our findings indicate that there is a gap in primary care provision for transgender clients in Northern Ontario including lack of gender-affirming services as well as a lack of knowledge in primary care practitioners.

The lack of gender-affirming services in Northern Ontario in our study was described as a ‘void’ with transgender individuals needing to travel great distances to seek care. Our findings are consistent with other studies that have noted a limited number of practitioners providing gender-affirming services in Canada, and patients are often on waiting lists or traveling to access care (Heinz and MacFarlane, 2013; Rotondi et al., 2013). However, this wait time is amplified in rural and remote communities of Northern Ontario. For routine care, residents of Northern Ontario are much farther from care compared with Southern communities, having to travel four times farther to access care (Tepper et al., 2006).

Access to gender-affirming services has been identified as a priority in the transgender population (Heinz and MacFarlane, 2013; Bourns, 2019). The value of providing access to gender-affirming services in primary care has been well documented (Cosio et al., 2022; Ker et al., 2020; Ross et al., 2016; Ziegler et al., 2019), with a need for integration of these services into more primary care practices. Participants suggested they could easily integrate primary care for transgender patients into their work. Prior research with primary care practitioners in Southern Ontario found similar results indicating the provision of primary care to transgender individuals was routine and part of their everyday practice (Ziegler et al., 2019). However, participants in this study noted challenges to integrate other colleagues into the work and oftentimes were the lone practitioner working with transgender patients.

Northern Ontario practitioners in our study indicated a lack of knowledge about gender-affirming care in their primary care settings. This lack of knowledge about LGBTQ healthcare needs in practitioners also persists as a dominant concern within other rural areas in Canada (Henriquez and Ahmad, 2021). When knowledge was not available in their primary care settings, practitioners took it upon themselves to learn how to provide care. Previous literature highlights how transgender individuals had to teach their healthcare practitioners about transgender-specific health issues as their practitioners did not have this knowledge (Bauer et al., 2009; Bauer et al., 2014; Heinz and MacFarlane, 2013), as limited content was covered in medical and nursing curriculum (Chan et al., 2016; Dubin et al., 2018; Lim et al., 2015; Nolan et al., 2020). A Canadian study found physicians and nurses were uncertain about transgender care and wanted more specialized knowledge and education (Beagan et al., 2013). The lack of formal and continuing education on transgender care is also a barrier. In a study by Ziegler et al. (2020b), participants identified key resources such as clinical practice guidelines, transgender-specific training and conferences as main opportunities for professional development. Furthermore, professional development training increases knowledge, competence, and confidence levels of healthcare practitioners caring for transgender individuals (Burgwal et al., 2021). In a systematic review, access to high-quality continuing education was found to be an important intervention in retaining health practitioners in rural and remote regions of Canada (Lafortune and Gustafson, 2019). The lack of continuing education with regard to transgender care in an underserved region is particularly notable.

### Implications

Both qualitative and quantitative findings of this study underscore a number of significant implications for practice, research and policy.

Practice implications suggest that practitioners in rural Canadian communities, particularly in Northern Ontario, need professional development opportunities to support service provision for transgender clients. Additionally, given the large population of Indigenous people living in Northern Ontario, training non-Indigenous practitioners about Indigenous and Two-Spirit identities and having Indigenous practitioners available could help mitigate fear and distrust and potentially increase access to primary care. There is also a need to tailor resources and outreach programs to meet the needs of Two-Spirit and gender-diverse Indigenous people, since strategies applied to the transgender population may not be relevant or effective to them (Bourns, 2019). Innovative and collaborative practices that incorporate virtual care may address some of the geographic barriers and lengthy waitlists.

Research implications reveal there is a paucity of research on the primary care needs of transgender people in Canada and internationally. The research that does exist focuses on hormone and surgical needs and less on preventative and chronic care, mental health issues, or on providing inclusive, safe, holistic non-stigmatizing care (Almazan and Keuroghlian, 2021; Kellett and Fitton, 2017; Kuzma et al., 2019; Ziegler, 2020b). Future research could focus on addressing the lack of knowledge among primary care practitioners by piloting and evaluating gender-affirming professional development and educational programs for primary care practitioners in in Northern Ontario.

Policy implications indicate that leadership support within primary care settings in Northern Ontario is needed to develop transgender-specific health programs, including the allocation of financial resources to address gaps such as training (Vupputuri et al., 2020). Advocacy for increased primary care services for this population is vital. Policymakers at the provincial and federal levels need to be aware of the increased challenges and barriers to care faced by the transgender population, and how these barriers are amplified in the Northern remote and rural populations.

### Limitations

Implementation was explored retrospectively; therefore, results may be affected by recall bias. Participants in this study were already providing care to transgender individuals; therefore, the study may not be generalizable to healthcare settings that do not have transgender patients, or practitioners that do not have specialized knowledge of transgender healthcare needs. The experience of providing healthcare in Northern Ontario may not be generalizable to more urban areas. The small sample size is likely not representative of the primary care workforce, which may limit the generalizability of the findings. In addition, the majority of the sample was representative of the nursing workforce also may not be reflective of those providing primary care to transgender individuals, which could limit the findings of this study.

The voice of the Indigenous, First Nations, or Métis transgender or Two-Spirit populations was not captured in this study highlighting another limitation. None of the practitioners worked specifically with the Indigenous, First Nations, or Métis populations. Despite these limitations, results could be used to inform other healthcare settings interested in providing primary care services to the transgender population.

### Conclusion

This study helps us to start to understand how primary care services are implemented and delivered in Northern Ontario. Despite the great efforts and strides made by participants in this study to improve access to care, barriers still exist. There continue to be limited practitioners providing this care, lack of access to gender-affirming healthcare services, lack of knowledge, geographic boundaries, and waitlists. More primary care practitioners are needed to embed these primary care services into their practice to improve access to gender-affirming care in Ontario.
